# Obstetric critical care: A prospective analysis of clinical characteristics, predictability, and fetomaternal outcome in a new dedicated obstetric intensive care unit

**DOI:** 10.4103/0019-5049.79895

**Published:** 2011

**Authors:** Sunanda Gupta, Udita Naithani, Vimla Doshi, Vaibhav Bhargava, Bhavani S Vijay

**Affiliations:** Department of Anaesthesiology and Critical Care, R.N.T. Medical College, Udaipur, India

**Keywords:** Critical care, MPM II score, obstetric ICU, outcome analysis, prediction of maternal mortality

## Abstract

A 1 year prospective analysis of all critically ill obstetric patients admitted to a newly developed dedicated obstetric intensive care unit (ICU) was done in order to characterize causes of admissions, interventions required, course and foetal maternal outcome. Utilization of mortality probability model II (MPM II) at admission for predicting maternal mortality was also assessed. During this period there were 16,756 deliveries with 79 maternal deaths (maternal mortality rate 4.7/1000 deliveries). There were 24 ICU admissions (ICU utilization ratio 0.14%) with mean age of 25.21±4.075 years and mean gestational age of 36.04±3.862 weeks. Postpartum admissions were significantly higher (83.33% n=20, *P*<0.05) with more patients presenting with obstetric complications (91.66%, n=22, *P*<0.01) as compared to medical complications (8.32% n=2). Obstetric haemorrhage (n=15, 62.5%) and haemodynamic instability (n=20, 83.33%) were considered to be significant risk factors for ICU admission (*P*=0.000). Inotropic support was required in 22 patients (91.66%) while 17 patients (70.83%) required ventilatory support but they did not contribute to risk factors for poor outcome. The mean duration of ventilation (30.17±21.65 h) and ICU stay (39.42±33.70 h) were of significantly longer duration in survivors (*P*=0.01, *P*=0.00 respectively) versus non-survivors. The observed mortality (n=10, 41.67%) was significantly higher than MPM II predicted death rate (26.43%, *P*=0.002). We conclude that obstetric haemorrhage leading to haemodynamic instability remains the leading cause of ICU admission and MPM II scores at admission under predict the maternal mortality.

## INTRODUCTION

Care of the critically ill parturients is a unique challenge in obstetrics particularly because of its unpredictability. Haemorrhage, toxaemia, anaemia and septicaemia are common causes of mortality and morbidity in these patients.[1]

Obstetric critical care in developing countries, however, continues to be radically different from developed countries.[2] An efficient scoring system for assessment of the severity and outcome in the critically ill obstetric patients would not only contribute to the assessment of the quality of patient care but would also enhance the risk stratification of pregnant patients in the evaluation of new therapies.[3] Various scoring systems like simplified acute physiology score (SAPS), acute physiology and chronic health evaluation (APACHE), and mortality probability models (MPM) have been used to predict the outcome of obstetric patients in the developed world[4] but ICUs from the Indian subcontinent seldom ever participated in these studies as a dedicated ICU for obstetric patients is not yet widely available in developing countries.[5,6]

With this background a 1 year prospective analysis of all critically ill obstetric patients admitted to a dedicated obstetric ICU was done to characterize the causes, clinical course, treatment, and foetal maternal outcome. Utility of MPM II score to predict maternal mortality was also assessed.

## METHODS

A 1 year prospective analysis of all obstetric admissions to the three-bedded new dedicated obstetric ICU at a women’s hospital attached to a tertiary care centre was conducted from 1 April 2009 to 31 March 2010, following ethical committee approval. Patients included were critically ill women admitted during pregnancy as well as in first 6 weeks of the postpartum period.

The critical care team included resident doctors from anaesthesiology (1), obstetrics (1) and a nurse in 8 hourly shifts. Medical and surgical consultants were available on call.

Admission criteria in obstetric ICU: Critically ill obstetric patients requiring ventilatory support or major organ supportive therapy were admitted to the obstetric ICU.

Facilities: Our obstetric ICU is divided into a clean area with two ICU beds and a septic area with one ICU bed located in vicinity of labour room and near the operation theatre complex. Major equipments include three L and T multiparameter monitors (electro cardio gram (ECG), non invasive blood pressure (NIBP)/ invasive blood pressure (IBP), heart rate (HR), oxygen saturation (SpO_2_), respiratory rate, temperature), microprocessor controlled ventilator with weaning modes (Nelcor Puritan Bennett) for each bed, crash cart, defibrillator, suction machine and electrocardiographic machine.

Data collection: An exhaustive proforma was developed to record the various data of patients admitted to obstetric ICU. The data collected included basic demographic data, obstetric and medical history, status before hospital admission, hospital course, ICU course, treatment taken and the specific interventions done. Data of total obstetric mortalities and total deliveries during the 1 year period to calculate maternal mortality per 1000 deliveries were also noted from the hospital administration system. Basic demographic data included literacy levels (uneducated: cannot read or write) and antenatal care (provided: completed three antenatal visits). The distance travelled was used as < / > 50 km as the hilly terrain and the poor transport system of this tribal area result in nearly 2 h of travel to cover a distance of 50 km.

Scoring tool: In all ICU-admitted obstetric patients, MPM II score at the time of admission was calculated to assess the ICU outcome in terms of predicted death rate.[4]

A receiver operator characteristic (ROC) curve was generated for MPM II at admission. The ROC curve represents a graphic display of sensitivity plotted against 1–specificity. Sensitivity was taken on ‘*y*’ axis and 1–specificity was plotted on ‘*x*’ axis. The criterion value for MPM II admission was considered positive for non-survivors and negative for survivors. The accuracy was measured by the area under the ROC curve. MPM II was also assessed at 24, 48 and 72 h during their stay in ICU.

Data analysis: All obstetric admissions were analyzed for their indications of admission, complications, associated medical conditions, duration of stay and interventions carried out in the ICU, and maternal and foetal outcome. Data were analyzed using the Statistical Package for Social Sciences (version 13, SPSS, Chicago, IL). For normally distributed demographic data, results were given as mean and standard deviations (SD). The Student *t*-test was used to compare mean variables in survivors and non-survivors. The chi-square test was used to compare categorical variables in survivor and non-survivor groups. A ‘*P* value’ of less than 0.05 was considered significant.

## RESULTS

In the 1 year period from 1 April 2009 to 31March 2010, 16,756 women delivered in our hospital, with 79 maternal deaths, giving a maternal mortality ratio of 4.7/1000 deliveries. The total admissions in the obstetric ICU were 24 women (ICU utilization rate was 0.14 per 100 deliveries) with 14 (58.33%) survivors and 10 (41.67%) non-survivors. The mean age of the patients was 25.21±4.075 years and the mean gestational age was 36.04±3.862 weeks. Demographic details of 24 patients according to maternal outcome are shown in Table 1. No demographic data were found as a risk factor for maternal mortality (*P*>0.05).

**Table 1:** Demographic characteristics according to maternal outcome

Characteristic feature		Total (%)	Survivors (%)	Non-survivors (%)	*P* value
Number of patients		24	14 (58.33)	10 (41.67)	0.420
Age (in years) Mean age (years) = 25.21±4.075	≤ 20	2 (8.33)	2 (8.33)	0	0.153
	21 – 30*	18 (75)	18 (75)	8 (33.33)	0.348
	≥31	4 (16.67)	2 (8.33)	2 (8.33)	0.305
Literacy	Educated	11 (45.83)	7 (29.16)	4 (16.6)	0.128
	Uneducated	13 (54.16)	7 (29.16)	6 (25)	0.688
Background	Rural	13 (54.16)	9 (37.5)	4 (16.66)	0.162
	Urban	11 (45.83)	5 (20.83)	6 (25)	0.688
Antenatal care	Provided**	19 (79.16)	11 (45.83)	8 (33.33)	0.499
	Not provided	5 (20.83)	3 (12.5)	2 (8.33)	0.642
Parity	P_1_	13 (54.16)	8 (33.3)	5 (20.83)	0.410
	P_2_	7 (29.16)	3 (12.5)	4 (16.67)	0.672
	P_3_	4 (16.67)	3 (12.5)	1 (4.16)	0.319
Distance	< 50 km	16 (66.67)	11 (45.83)	5 (20.83)	0.130
	> 50 km	8 (33.3)	3 (12.5)	5 (20.83)	0.487

* Data are expressed as n (%), *P = 0.000

** *P* = 0.005

Postpartum admissions (n=20, 83.33%) were significantly more as compared to antepartum admissions (n=4, 16.66%, *P*<0.05). Obstetric complications (n=22, 91.66%) were a significant cause of severe morbidity as compared to non-obstetric (medical) complications (n=2, 8.34% *P*<0.01) of which obstetric haemorrhage (n=15, 62.5%) was found to be a significant risk factor for ICU admission, (*P*=0.000) [Table 2].

**Table 2:** Diagnosis of patients admitted to intensive care unit

Complications	Diagnosis	No. of patients n = 24	Admitted from OT n = 13 (54.16%)		Admitted from wards n=11(45.83%)	*P* value
Obstetric complications (n=22, 91.66%)	Obstetric haemorrhage*	15 (62.5)	10 (41.66)	C=1 (4.16);	5 (20.83)	0.194
				H=2 (8.32);		
				C+H=7 (29.16)		
	Pregnancy induced hypertension†	4 (16.66)	0		4 (16.66)	0.318
	Pulmonary embolism	1 (4.16)	0		1 (4.16)	0.318
	Septicaemia	2 (8.33)	2 (8.33) (C)		0	0.153
Non-obstetric complications (n=2, 8.34%)	Pulmonary oedema	2 (8.33)	1 (4.16) (C)		1 (4.16)	0.305

* Data are expressed as n (%), *P* = 0.000, C = Caesarean section, H = Hysterectomy

† n = 4 (antepartum), (C+H) = Caesarean hysterectomy, OT= Operation theatre

Some of the associated medical conditions included nutritional anaemia (n=8, 33.33%, *P*= 0.010), jaundice (n=2, 8.33%), mitral valve disease (n=1,4.16%) and upper respiratory tract infection (URTI) (n=1,4.16%) in 12 patients. This patient of URTI was already septicaemic with hemodynamic instability, who underwent emergency caesarean section and was directly shifted to the obstetric ICU where she expired after 24 h due to septicaemic shock [Table 3].

**Table 3:** MPM II predicted death rate at various time intervals, along with indications and outcome

Diagnosis	Associated medical conditions	MPM II predicted death rate at				No. of ICU hours	Ventilatory support	Indications (type of resp failure	Outcome of ICU stay	Cause of death
						
		Admission	24hr	48hr	72hr		Yes/No	I/II/III/IV		
Obstetric haemorrhage		6.2	1.6	W		30	Yes	IV	Survivor	
Obstetric haemorrhage		18	4.9	2.5	W	72	Yes	IV	Survivor	
Antepartum eclampsia (PIH)		1.7	3.0	4.3	9.74	144	Yes	II	Survivor	
Obstetric haemorrhage		11	W			24	No		Survivor	
Septicemia	URTI	13	49.0	D		24	Yes	I	Non survivor	SSS
Pulmonary embolism		34.6	D			24	Yes	I	Non survivor	MODS
Obstetric haemorrhage		43.3	D			24	Yes	IV	Non survivor	MODS
Obstetric haemorrhage	Anemia	4.2	W			24	No		Survivor	
Obstetric haemorrhage	Anemia	47.1	D			24	Yes	IV	Non survivor	DIC
Antepartum eclampsia (PIH)	Anemia	16	D			72	Yes	II	Non survivor	MODS
Obstetric haemorrhage	Anemia	28.9	D			24	Yes	IV	Non survivor	MODS
Obstetric haemorrhage	Anemia	43.3	4.7	W		24	No		Survivor	
Pulmonary oedema	MS/MR	2.8	W			24	Yes	I	Survivor	
Obstetric haemorrhage	Anemia	43.3	18.4	W		24	Yes	IV	Survivor	
Pulmonary oedema		28.8	D			24	Yes	I	Non survivor	MODS
Obstetric haemorrhage		11	11.9	D		48	No		Non survivor	MODS
Obstetric haemorrhage	Anemia	92.1	D			24	Yes	IV	Non survivor	MODS
Obstetric haemorrhage		11.6	1.6	W		48	No		Survivor	
Antepartum eclampsia (PIH)	Anemia	8.2	7.2	9.0	W	48	Yes	II	Survivor	
Septicemia	Jaundice	53.9	D			24	Yes	I	Non survivor	MODS
Obstetric haemorrhage		24.7	8.6	2.6	W	48	No		Survivor	
Obstetric haemorrhage		56.1	30.7	7.2	W	72	Yes	IV	Survivor	
Antepartum eclampsia (PIH)		10.2	3.1	W		48	No		Survivor	
Obstetric haemorrhage	Jaundice	24.2	20.5	25	W	48	Yes	IV	Survivor	

ICU = Intensive care unit, W = Shifted to ward, PIH = Pregnancy induced hypertension, D = Death, SSS = septic shock syndrome, MODS = multi organ dysfunction, DIC = disseminated intravascular coagulopathy, URTI = upper resp inf

**Figure 1:**
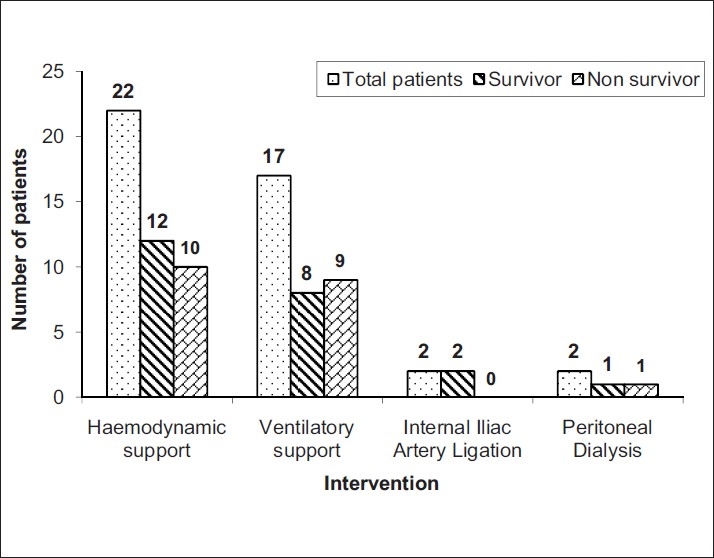
Distribution of patients according to interventions given and maternal outcome

When primary indications for ICU admission were analysed, haemodynamic instability (n=20, 83.33%) was the most common and significant cause of admission to ICU as compared to respiratory insufficiency (n=3, 12.54%) and neurological dysfunction (n=1, 4.16%), *P*=0.000. However, during the course of treatment 22 (91.66%) patients required inotropic support (vasopressors) and 17 (70.83%) patients required ventilatory support, but these interventions were not found to be a significant risk factor for mortality (*P*>0.05) [Figure 1].

Out of the 17 patients requiring ventilator support there were 8 (47.05%) survivors and 9 (52.94%) non-survivors. The mean duration of ventilation was 30.17±21.65 h (range 0.5–96 h) with survivors having significantly longer duration of controlled ventilation (41.14±28.54 h), as compared to non-survivors (20.56±22.25 h, *P*=0.01).

The mean duration of stay in the ICU was 39.42±33.70 h (range 2-144 h) with significantly longer duration of stay in survivors (50.86±36.6 h), as compared to non-survivors (23.40±21.681 h, *P*=0.000).

**Figure 2:**
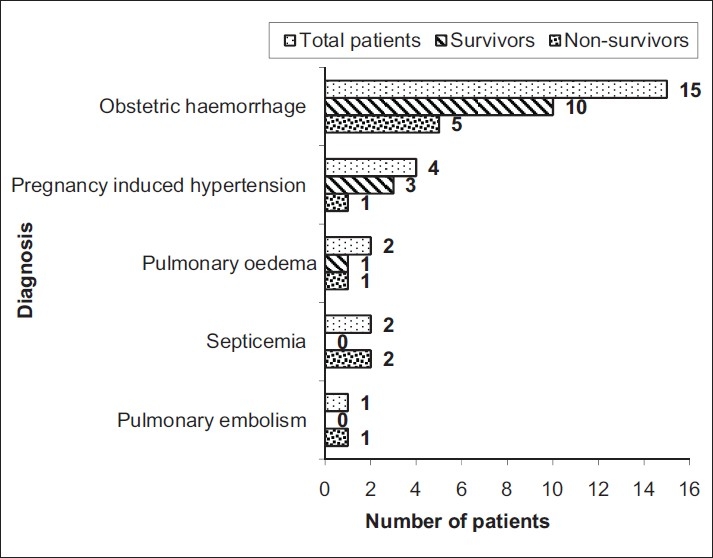
Distribution of patients according to maternal outcome in ICU (n)

As can be seen in Figure 2, maternal outcome according to patient diagnosis shows that obstetric haemorrhage (n=15, 62.50%) was a significant cause for ICU admission (*P*=0.000) but none of the diagnosis was found to be a significant risk factor for maternal mortality (*P*>0.05). Multi-organ dysfunction syndrome (MODS) (n=8, 80%) was found to be the most significant (*P*=0.008) cause of maternal mortality, while other causes were disseminated intravascular coagulation (DIC) (n=1, 10%) and septic shock syndrome (n=1, 10%).

The mean predicted death rate as calculated by MPM II at admission was 26.43±21.9 (range 1.7-92.1). The predicted death rate was significantly higher for the non-survivors (36.87±24.25) as compared to the survivors (18.96±17.26, *P*=0.046). The observed mortality was 41.67% (n=10), which was significantly higher than predicted death rate obtained by MPM II (26.43%, *P*=0.002). The ratio of observed mortality to predicted death rate was 1.57 indicating that MPM II score had under-predicted the mortality in these patients. Table 3 shows the distribution of patients according to the MPM II predicted death rate at various time intervals along with their outcome. A progressive rise in predicted death rate was seen in most of the non-survivors and a fall was seen in most of the patients who were shifted to the wards after stabilization.

**Figure 3:**
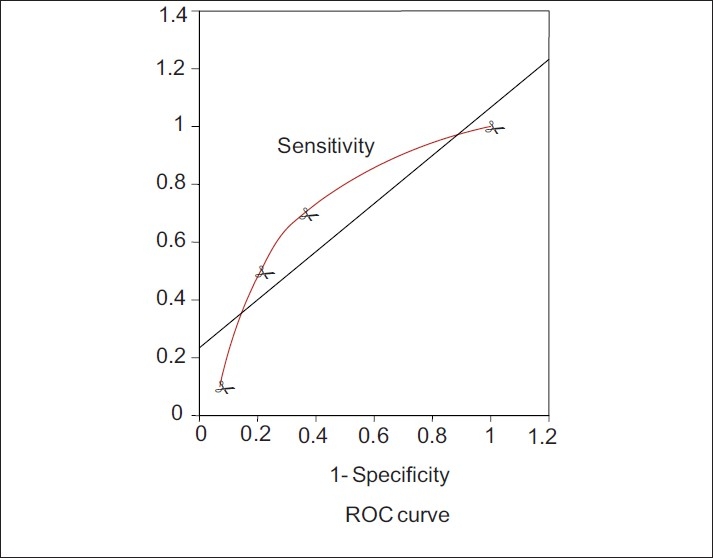
Receiver operator characteristic curve (ROC curve) of MPM II score at admission for obstetric patients

When the ROC curve was generated for MPM II, the area of distribution under the ROC curve was fair, i.e. 0.74 [Figure 3]. The ROC curve could not be generated at 24 h, 42 h and 72 h since number of patients in ICU at these time intervals were decreased.

## DISCUSSION

Clinical recognition of the unique needs of the critically ill obstetric patients have received much attention in an attempt to assess the need for dedicated critical care facilities.[7,8] Since, in general, for most obstetric patients, rapid recovery follows correction of the acute insult. It is now believed that between 0.1% and 0.9% of parturients have complications requiring ICU admission.[9] On analysis of the critically ill obstetric patients in our hospital for a year, we found a dismal ICU utilization rate (n= 24, 0.14%) in spite of the high maternal mortality (n= 79, 4.7/1000 deliveries). Since most of the mortality (n= 69/79, 93.24%) occurred in wards or the emergency outpatient areas without utilizing ICU services, a delay in identification of criticality of such patients could be a major cause for under utilization of the ICU. The demographic details and causes of mortality in these patients have not been identified, as our study population included only patient admitted to obstetric ICU. A higher utilization of ICU services in the developed countries has been observed (0.70,[4] 0.76,[10] 0.90,[11] 1.40[5]).Obstetric patients are usually young but the gestational age of critically ill parturients shows a variance in different studies.[11–13] In our study most of the parturients at term (36 weeks) were admitted for obstetric haemorrhage, while in the studies from developed countries, they were admitted for pre-eclampsia (29 weeks,[11] 29.6[12] weeks, 31.7 weeks[13]) which could explain this difference in gestational age.

Low socioeconomic status, lack of education and poor antenatal care have been found to have a considerable effect on obstetric complications and outcome.[5] However, we could not find any association of factors like level of literacy, rural/ urban background, and distance travelled for reaching the hospital with higher incidence of ICU admission or poor outcome. The lack of antenatal care has not been associated as a risk factor for ICU admissions[5,11,13] as was also observed in our study.

It has been reported[14,15] that the most common reasons for ICU admission for obstetric patients are hypertensive disorders and massive obstetric haemorrhage. It was emphasized that early detection and prompt referral to tertiary centres with intensive care facilities to provide optimum care of circulation, blood pressure and ventilation could minimize the prevalence of multiple organ failure and mortality in critically ill obstetric patients. Our study group revealed a higher rate of obstetric rather than medical complications. As reported by other studies,[4,6,13,16] we observed that obstetric haemorrhage was the major cause for ICU admission. In our series, it represented the main cause for ICU admission (62.5%). At the same time, in comparison with other authors,[4,5,10,13,17] we found a lower percentage of pre-eclampsia and eclampsia.

Most of the authors have reported a higher incidence of postpartum admission to the obstetric ICU (100%,[5,17] 91%,[13] 78%,[18] 66%[12]) as was also seen in our study (83.3%). This could be attributed to the haemodynamic changes in the postpartum period which shows a 65% increase in cardiac output, acute blood loss during delivery and decrease in plasma oncotic pressure.[19] Secondly there is general reluctance to move a pregnant woman away from the proficiency of obstetrician’s care unless it is absolutely necessary. Though hemodynamic instability can usually be managed in the labour room area, but the need for mechanical ventilation remains the major indication for antenatal ICU transfer. After delivery, criteria for ICU transfer become generalized since services of obstetricians are no more a priority.[12] In our study, ventilator support was a major indication for ICU admissions among antenatal patients with pregnancy-induced hypertensive disorders (n=3/4, 75%).

It has been observed that hemodynamic and respiratory complications needing inotropic or ventilator support remain the most common reasons for ICU admissions[12,13] and the need for support may predict poor outcome.[5] In the present study, 91.66% patients required inotropic support and 70.83% required ventilatory support. Although not statistically significant, the association of mortality with both these supports was considerable (10/22 and 9/17 respectively). The mean duration of ventilation and ICU stay was apparently less in our study than others,[12,20–22] which could be attributed to the higher mortality rate (41.67%) in our study. Incidence of maternal mortality has significantly decreased in the developed countries (0%,[13] 11%,[11] 27.78%[17]) as compared to the developing countries (50%,[5] 40.35%[6]). Increased maternal mortality rates in developing countries have been attributed to treatment by quacks, low socio-economic status, non-existent antenatal care, low haematocrit and under-nourishment in obstetric patients.[6] Our maternal mortality was 41.67%, but there was no association of any of the above demographic factors with the incidence of mortality. We found multi-organ failure including heart failure, shock lung and acute renal failure to be the leading cause of maternal mortality (80%) as reported earlier.[11]

It has been observed that if antepartum patients have viable foetus and delivered or underwent caesarean section, while under intensive care there would not be higher incidence of neonatal mortality.[12] In our study there were four antenatal patients pregnancy induced hypertension (PIH) with intrauterine death and delivery was conducted in ICU with no foetal survivals (0%). Patients who were admitted with obstetric haemorrhage (n=15) had significantly better foetal survival (n=12 (80%), *P*=0.019).

The paucity of critically ill obstetric patients in any population used to validate scoring systems precludes the routine use of such system in this subset of patients. It has been observed that when obstetric patients are admitted for medical disorders, the predicted mortality rate (PMR) correlates with the observed mortality rate (OMR). However in patients with obstetric disorders, the OMR is much lower than the PMR. APACHE and SAPS II scoring systems likewise overestimate mortality in obstetric patients.[10,11,15] This overestimation of the risk could be attributed to reversibility of certain obstetric pathologies like preeclampsia and haemorrhage if there is effective and timely management. Further, some of the criteria used for these scoring systems are based on physiological changes of pregnancy rather than on pathological changes of pregnancy.

After analysis of the model performance of MPM II, APACHE II, SAPS II scores it has been declared that if the area the under ROC curve is in the neighbourhood of 0.5, the model is performing no better than coin toss. Developers of models are typically not satisfied unless area of ROC of a model exceeds 0.7.[23] We had a fair (i.e. 0.74) area of distribution under the ROC curve for MPM II. Actual mortality (41.67%) was significantly higher than MPM II predicted mortality (26.43) indicating that MPM II has underestimated the mortality or performance of our ICU was substandard. However, in a retrospective record review,[4] the predictive ability of APACHE II, SAPS II and MPM II scores in critically ill obstetric patients was evaluated and compared to a control group of non-obstetric female patients of similar age groups (17 to 41 years) who were admitted to medical and surgical ICUs, the observed mortality was not statistically different from the mortality predicted by APACHE II, SAPS II and MPM II for the obstetric group and non-obstetric group and predictive accuracy of these models as assessed by the c-index, which is equivalent to the area under the ROC curve has been proved.

The ratio of observed death to the expected number of deaths can vary from 0.67 to 1.21 (APACHE II[24]), or 0.74 to 1.31 (SAPS II[25]). It has been suggested that it does not necessarily reflect the performance of ICU to be below or above par when the ratio is >1 or <1.[23] To interpret these ratios effectively, one should also consider and identify factors that are associated with the observed and expected mortality differential, as their probabilities, by themselves, do not control for all of the differences that may have an impact on outcome.

The major limitation of our study being a single centre study was that it was not feasible to validate our conclusions as the sample size was small (n=24). A potential selection bias in our study could have been avoided, if demographic detail and cause of mortality (n=69) that occurred outside the ICU during the period of study had been identified.

## CONCLUSIONS

In spite of limitations, some careful conclusions can be drawn. We conclude that obstetric haemorrhage leading to haemodynamic instability remains the leading cause of ICU admission. Inotropic support and ventilatory support are the main interventions provided in the ICU which were not found to be associated with poor outcome. Duration of ventilation and stay in the ICU were significantly more in the survivors.

Accurate predictive scores in the ICUs apart from providing aggressive management in those predicted for a poor outcome, could also lead to better productive utilization of the limited resources. Our results, using the MPM II (admission) scores were found to under-predict the mortality, highlighting the importance of a continued search for better scoring system in the critically ill obstetric patients.

In future multicenteric studies, focusing on audit of obstetric ICUs in India will help to validate such observations as found in our study. It will also improve patient care and stimulate education in the management of such patients among the resident doctors, consultants and nursing staff. A better scoring system especially applicable to the critically ill obstetric patients in the Indian scenario could lead to accurate monitoring of quality care and risk stratification for clinical and therapeutic trials.
